# Efficacy and Safety of Pericapsular Nerve Group Block for Hip Fracture Surgery under Spinal Anesthesia: A Meta-Analysis

**DOI:** 10.1155/2024/6896066

**Published:** 2024-03-13

**Authors:** Shukai Li, Jing An, Chengyu Qian, Zhixue Wang

**Affiliations:** Department of Anesthesiology, Affiliated Hospital of Chengde Medical University, Chengde 067000, China

## Abstract

**Objective:**

To evaluate the effectiveness and safety of pericapsular nerve group (PENG) block for hip fracture surgery under spinal anesthesia.

**Methods:**

This meta-analysis was registered on INPLASY (INPLASY202270005). PubMed, Embase, Cochrane, CNKI, and Wanfang databases were searched to collect the randomized controlled trials of the PENG block applied to hip fracture surgery in the setting of spinal anesthesia, with the search period from inception to 1 May 2023. Two independent researchers gradually screened the literature, evaluated the quality, extracted the data, and eventually pooled data using RevMan 5.4.

**Results:**

Fifteen articles with 890 patients were enrolled. The combined results showed that the PENG block reduced pain scores during position placement (SMD = −0.35; 95% CI [−0.67, 0.02]; *P*=0.04; *I*^2^ = 0%). Subgroup analyses showed that compared to the unblocked group, the PENG block reduced pain scores at 12 h, 24 h, and 48 h postoperatively. The incidence of postoperative hypokinesia was reduced (RR = 0.11; 95% CI [0.01, 0.86]; *P*=0.04; *I*^2^ = 0.00%). The time to first walking was advanced (SMD = −0.90; 95% CI [−1.17, 0.63]; *P* < 0.00001; *I*^2^ = 0%).

**Conclusion:**

The PENG block can reduce postoperative pain and pain during spinal anesthesia positioning, which is helpful to improve the operability and comfort of spinal anesthesia and facilitate postoperative muscle strength recovery and early activity.

## 1. Introduction

Hip fracture is one of the standard clinical fractures, and its incidence has increased significantly, especially among people over 65 years old. Hip fracture is a severe trauma due to osteoporosis and trauma, etc. It is accompanied by severe pain, complications can be disabling or even life-threatening, and surgical treatment is used as its primary treatment [1]. Spinal anesthesia is still one of the most commonly used methods [2]. However, severe pain caused by fractures often leads to difficulties in placement and positioning of the spinal anesthesia position, making spinal anesthesia more challenging to perform, and poor management of postoperative pain and other complications can significantly impact the patient's early recovery [3]. Therefore, finding an effective solution to synergize spinal anesthesia is essential.

Recently, the pericapsular nerve group (PENG) block of the hip joint has gained increasing attention, first described by Giron-Arango et al. The PENG block, usually performed under ultrasound guidance, is performed by injecting local anesthetic drugs to block the articular branches of the femoral nerve (FN), the obturator nerve (ON), and the accessory obturator nerves (AON), among others. These nerves provide sensory innervation to the anterior hip capsule [4] to achieve its analgesic effect. The PENG block has been reported to have the advantages of easy operation, fewer complications, better postoperative analgesia, and the ability to be performed in the supine position, and it can reduce pain without affecting motor function [5]. Therefore, patients undergoing the PENG block can get out of bed as early as possible, reducing the incidence of postoperative complications and facilitating early recovery [6].

Although some clinical studies have reported the application of the PENG block in hip fracture surgery, there is no conclusion about its effectiveness and safety for hip fracture surgery under the circumstances of spinal anesthesia. In this study, we conducted a meta-analysis of published high-quality RCTs to systematically evaluate the effectiveness and safety of the PENG block for hip fracture surgery under spinal anesthesia and to provide evidence-based medical evidence and reference basis for clinical practice.

## 2. Methods

### 2.1. Search Methods

**Figure 1:**
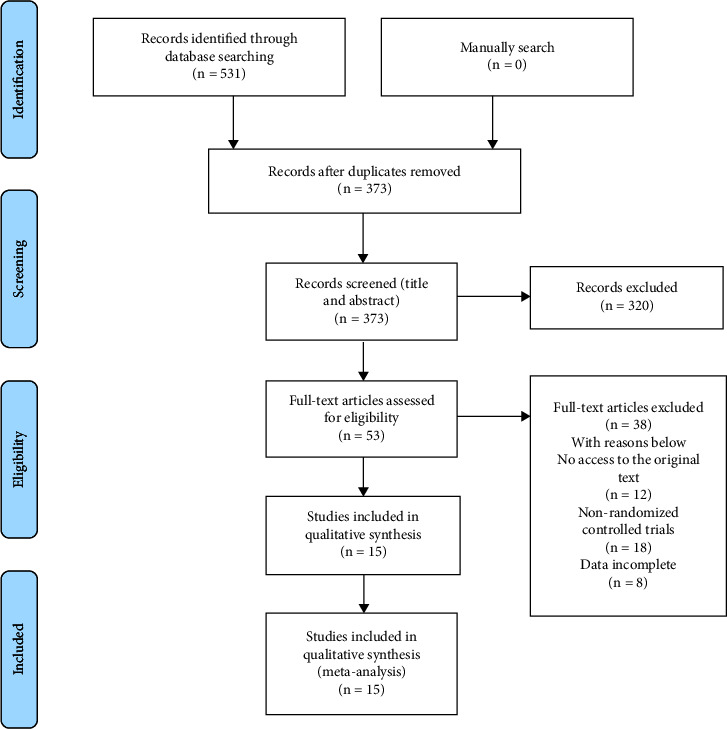
Study flowchart.

This meta-analysis was registered on INPLASY (INPLASY202270005). Our meta-analyses were performed according to PRISMA reporting standards. The search strategy was a comprehensive search of PubMed, Cochrane, Embase, CNKI, and Wanfang databases. The essential English search terms were “PENG block,” “Pericapsular Nerve Group block,” “Femoral Neck Fracture,” and “hip fracture.” Randomized controlled trials on the PENG block were collected. The search date was from the establishment of the database to 1 May 2023, and the search languages were English and Chinese, using a combination of subject terms and free words and adjusted according to the characteristics of each database. The references of the included studies were also searched to obtain additional relevant information. Detailed methodology can be referred to previous words [7–10]. The detailed search strategy is shown in Figure 1.

### 2.2. Study Selection and Data Collection

The initial retrieved literature was imported into Endnote 20, duplicates were excluded, and two researchers independently screened the literature, extracted the information, and cross-checked it; in case of disagreement, a third researcher was consulted to assist in the adjudication, and the lack of information was replenished by contacting the authors as much as possible. The data extracted included authors, year of publication, time of nerve block operation, number of people in intervention and control groups, postoperative remedial analgesic medications, and adverse effects. Pain scores (including numerical analog scales NRS and VAS) at each postoperative time point, which were converted to VAS (1–10 cm) for different pain scales [11, 12]; opioid consumption at 24 and 48 hours postoperatively, which needed to be converted to an equivalent amount of morphine if it was not morphine [13]; time of the first postoperative remedial analgesia; time of the operation of the spinal anesthesia; the time of the first postoperative ambulation out of bed; and the incidence of postoperative nausea and vomiting. Details are provided in Table 1.

### 2.3. Risk of Bias Assessment

**Figure 2:**
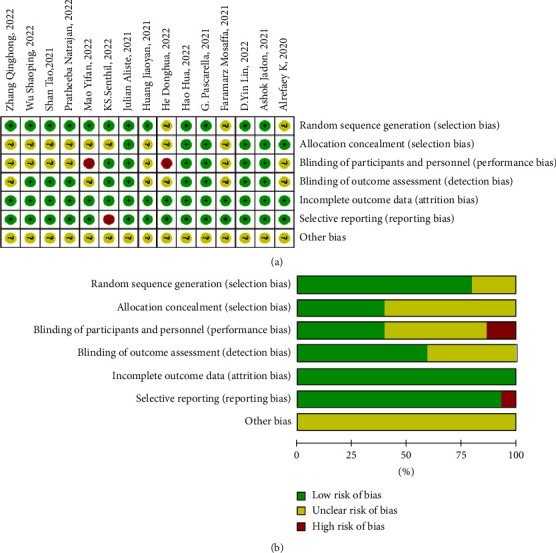
Risk of bias. (a) Risk of bias summary. Review authors' judgements about each risk of bias item for each included study. (b) Risk of bias graph. Review authors' judgements about each risk of bias item presented as percentages across all included studies.

Two independent researchers evaluated the included literature using the Cochrane Risk of Bias Assessment System. Evaluation entries included random sequence generation, allocation concealment, blindness of participants, outcome evaluators, completeness of outcome data, selective reporting of outcome indicators, and other biases. ReviewMan 5.4 was applied to map the risk of bias assessment, see Figure 2.

### 2.4. Statistical Analysis

Data were processed using RevMan 5.4. Continuous variables were described by the standardized mean difference (SMD) and 95% confidence interval (CI) and dichotomous variables by the relative risk ratio (RR). Heterogeneity between studies was evaluated using *I*^2^ values; with *I*^2^ ≤ 50%, no heterogeneity was considered to exist, and a fixed-effects model was selected; with *I*^2^ > 50%, significant heterogeneity was considered to exist, and a random-effects model was selected. Subgroup analyses (subgroup analyses were performed using different modes of basal analgesia in the control group) were used to find sources of heterogeneity.

### 2.5. Assessment of Evidence Quality

The GRADE profiler software was used to evaluate the quality of the evidence for the results of the combined analysis, and a high-, medium-, low-, or very low-quality evidence rating was made for each outcome, which was used to evaluate the quality of the evidence and the strength of the recommendations, as shown in Table 2.

## 3. Results

### 3.1. Results of the Search

The initial search yielded 531 articles, and fifteen articles were finally selected for inclusion after screening, including nine articles in English [2, 5, 15–20, 22] and six articles in Chinese [14, 21, 23–26]. A total of 890 patients, of which 449 were in the intervention group and 441 were in the control group, and the inclusion screening flowchart is shown in Figure 1.

### 3.2. Characteristics of Included Studies

Among all the included literature, there were fifteen articles in the intervention group in which the PENG block was performed, including eleven articles in which the PENG block was performed before spinal anesthesia and four articles in which the PENG block was performed after spinal anesthesia; a total of fifteen articles were included in the control group, in which no PENG block was performed, including seven articles in which the fascia iliaca compartment block (FICB) was performed (five in which FICB was performed before spinal anesthesia and two in which FICB was performed after spinal anesthesia) and eight in which no neural block was performed. Outcome metrics were reported in eight papers that reported time to first postoperative remedial analgesia, three papers that reported time to first postoperative ambulation, two papers that reported time to spinal anesthesia manipulation, a total of six papers that reported on the occurrence of postoperative hypokinesia, and eight papers that reported on the incidence of PONV. Four literature results were reported as the median and interquartile range (IQ range), which were converted according to the appropriate formulas. There was one literature with results reported in a graphical form. The essential characteristics of the included studies are tabulated in Table 1.

### 3.3. Methodological Quality Assessment

According to the Cochrane Risk of Bias Assessment System, among the literature we included, there were eight high-quality papers [5, 15, 17, 19–22, 25], three low-quality papers [2, 14, 24], and four papers with unclear literature quality [16, 18, 23, 26]. The results are shown in Figure 2.

### 3.4. Effects of Interventions

#### 3.4.1. Main Outcome Indicators

**Figure 3:**
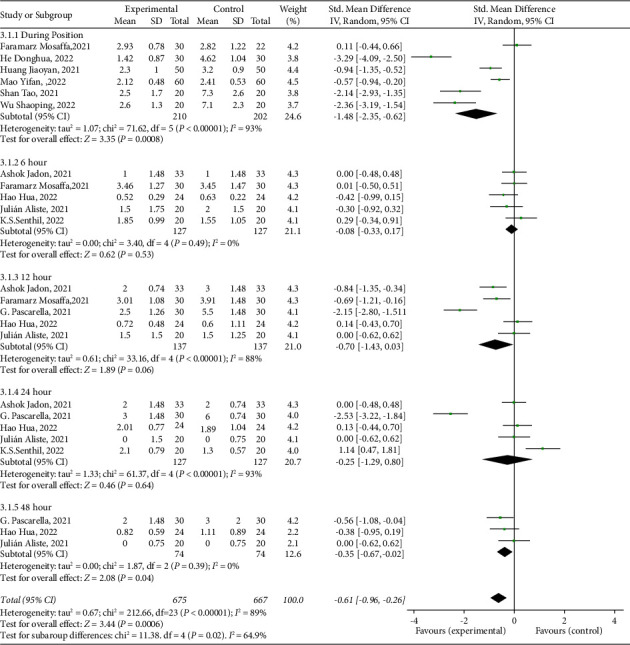
Forest of standardized mean differences in pain scores measured at different time points. The green square represents the effect of individual studies, and the vertical lines show the corresponding 95% confidence intervals (CIs). The black diamond reflects the overall or summary effect. The outer edges of the diamonds represent the CIs.

*(1) Pain Score during Position Placement (VAS)*. The pain score during position placement was defined as the patient's pain score during position placement for spinal anesthesia, and a total of six papers were included in this outcome index (SMD = −1.48; 95% CI [−2.53, 0.62]; *P*=0.0008; *I*^2^ = 93%, Figure 3). Pain scores were reduced in the PENG block group compared with the control group, and the difference was statistically significant (*P* < 0.05). After sensitivity analysis, excluding any of the studies did not change the direction of the results, indicating stable results. According to the subgroup analysis through the control group of different essential analgesia before spinal anesthesia (FICB subgroup before spinal anesthesia, no nerve block subgroup), the results are shown in Table 3. Compared with the FICB group before spinal anesthesia, the difference in pain scores between the PENG block group was not statistically significant; compared with the no nerve block group before spinal anesthesia, the pain scores of the PENG block group were reduced, and the difference was statistically significant (*P* < 0.05).

*(2) Postoperative Pain Scores (VAS)*. We extracted postoperative pain scores at 6 h, 12 h, 24 h, and 48 h. A total of 3–5 papers were included in this outcome metric, which was as follows: at 6 h postoperatively (SMD = −0.08; 95% CI [−0.33, 0.17]; *P*=0.53; *I*^2^ = 0%, Figure 3); at 12 h postoperatively (SMD = −0.70; 95% CI [−1.43, 0.03]; *P*=0.06; *I*^2^ = 88%, Figure 3); at 24 h postoperatively (SMD = −0.25; 95% CI [−1.29, 0.80]; *P*=0.64; *I*^2^ = 93%, Figure 3); and at 48 h postoperatively (SMD = −0.35; 95% CI [−0.67, −0.02]; *P*=0.04; *I*^2^ = 0%, Figure 3). Compared with the control group, the pain score was reduced in the PENG block group at 48 h postoperatively, and the difference was statistically significant (*P* < 0.05); the difference was not statistically significant at 6 h, 12 h, and 24 h postoperatively. The above was analyzed by sensitivity analysis, and excluding any of the studies did not change the direction of the results, indicating that the results were stable. According to the subgroup analysis through the control group of different primary analgesic modalities (FICB subgroup, not nerve block subgroup), the results are shown in Table 3. Compared with the FICB subgroup, the PENG block group postoperative pain scores of 6 h, 12 h, 24 h, and 48 h difference were not statistically significant; compared with the not nerve block group subgroup, the PENG block group postoperative pain scores of 12 h, 24 h, and 48 h were all were significantly lower, and the differences were statistically significant (*P*  <  0.05).

**Figure 4:**
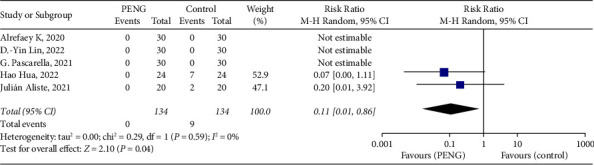
Forest of standardized mean differences in the incidence of postoperative hypokinesia. The blue square represents the effect of individual studies, and the vertical lines show the corresponding 95% confidence intervals (CIs). The black diamond reflects the overall or summary effect. The outer edges of the diamonds represent the CIs.

*(3) The Incidence of Postoperative Hypokinesia*. The incidence of postoperative hypokinesia was defined as postoperative knee or hip dyskinesia. A total of five papers were included for this outcome index, and the results were as follows: (RR = 0.11; 95% CI [0.01, 0.86]; *P*=0.04; *I*^2^ = 0.00%, Figure 4). The incidence of postoperative hypokinesia was reduced in the PENG block group compared with the control group, and the difference was statistically significant (*P* < 0.05). Subgroup analysis was performed according to the different primary analgesia modalities in the control group (FICB subgroup, no nerve block subgroup), and the results are shown in Table 4. Compared with the FICB subgroup, the PENG block reduced the incidence of postoperative hypokinesia, with a statistically significant difference (*P* < 0.05); compared with the no nerve block group subgroup, there was no statistically significant difference between the differences in the incidence of postoperative hypokinesia in the PENG block group.

#### 3.4.2. Secondary Outcome Indicators

*(1) Postoperative 24 h Opioid Consumption*. A total of six papers were included for this outcome (SMD = −1.27; 95% CI [−2.19, −0.35]; *P*=0.007; *I*^2^ = 92%), and the PENG blockade group had a statistically significant reduction in postoperative 24 h opioid consumption when compared with the control group (*P* < 0.05). After sensitivity analysis, excluding any of the studies did not change the direction of the results, indicating stable results. Subgroup analyses were performed by different primary analgesia modalities in the control group (FICB subgroup, no nerve block subgroup), and the results are shown in Table 5. Compared with the FICB subgroup, the PENG block could reduce 24 h postoperative opioid consumption, and the difference was statistically significant (*P* < 0.05); compared with the subgroup of the no nerve block group, the PENG block could reduce 24 h postoperative opioid consumption, and the difference was statistically significant (*P* < 0.05).

*(2) Postoperative 48 h Postoperative Opioid Consumption*. This result was included in a total of three papers; the results were as follows: (SMD = −0.33; 95% CI [−0.80, 0.15]; *P*=0.17; *I*^2^ = 59%). The inclusion of the literature control group is spinal anesthesia before the FICB, the PENG block could not reduce the 48 h postoperative opioid consumption, and the difference was not statistically significant.

*(3) The Time to First Postoperative Remedial Analgesia*. The time to first postoperative remedial analgesia, defined as the time from the end of the operation to the patient's first request for analgesic medication, was included in this result in eight papers. The time to first postoperative remedial analgesia (SMD = 1.38; 95% CI [0.41, 2.34]; *P*=0.005; *I*^2^ = 95%) was delayed in the PENG block group when compared to the control group, with a difference of statistical significance (*P* < 0.05). After sensitivity analysis, the exclusion of any of the studies did not change the direction of the results, indicating stable results. Subgroup analyses were performed by different primary analgesic modalities in the control group (FICB subgroup, unnerve block subgroup), and the results are shown in Table 6. Compared with the FICB subgroup, the PENG block group could not delay the time of the first postoperative remedial analgesia. The difference was not statistically significant, and compared with the unnerve block group subgroup, the PENG block group could delay the time of the first postoperative remedial analgesia. The difference was statistically significant (*P* < 0.05).

*(4) The Time of the spinal anesthesia operation*. The time of the spinal anesthesia operation was included in 2 papers, and the control group was without a nerve block. The results were as follows: (SMD = −1.29; 95% CI [−2.54, −0.03]; *P*=0.04; *I*^2^ = 84%), and the spinal anesthesia operation time was shortened in the PENG block group compared with the control group, with a statistically significant difference (*P* < 0.05). After sensitivity analysis, excluding any of the studies did not change the direction of the results, indicating that the results were stable.

**Figure 5:**
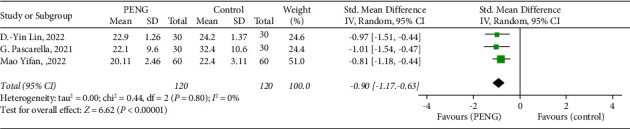
Forest of standardized mean differences in the time to first walking. The green square represents the effect of individual studies, and the vertical lines show the corresponding 95% confidence intervals (CIs). The black diamond reflects the overall or summary effect. The outer edges of the diamonds represent the CIs.

*(5) The Time to First Walking*. The time to first walking, defined as the time to first get out of bed after the end of the operation, was included in a total of three papers. The results were as follows: (SMD = −0.90; 95% CI [−1.17, −0.63]; *P* < 0.00001; *I*^2^ = 0%, Figure 5), the time to first walking was advanced in the PENG block group compared with the control group, and the difference was statistically significant (*P* < 0.05).

*(6) The Incidence of Postoperative PONV*. A total of eight papers were included, and the results were as follows: (RR = 0.67; 95% CI [0.34, 1.32]; *P* = 0.25; *I*^2^ = 0.00%), there was no difference of the incidence of postoperative nausea and vomiting between PENG blockade and control group, the difference was not statistically significant.

#### 3.4.3. Assessment of Evidence Quality

The quality of evidence for each indicator was evaluated using the GRADE profiler software. The results showed that there was no high-intensity evidence. Moderate-intensity evidence supports that the PENG block reduces postoperative 48 h pain, decreases the incidence of postoperative muscle strength reduction, and advances the time to first ambulation but does not reduce postoperative 6 h pain or the incidence of PONV. Low-equal-strength evidence supports that the PENG blockade does not reduce 48 h postoperative opioid consumption. Very low equal-intensity evidence supports that the PENG blockade reduces pain at the time of spinal anesthesia placement position at 12 h and 24 h postoperatively, reduces opioid consumption at 24 h postoperatively, delays the time to first remedial analgesia, and reduces the time to spinal anesthesia manipulation. The overall quality of the evidence was low, and there is a need to look for higher-quality evidence to demonstrate these points in upcoming studies.

## 4. Discussion

### 4.1. Summary of Main Results

A total of fifteen studies were included in this meta-analysis to analyze the effects of the PENG block in spinal anesthesia for hip fracture surgery on spinal anesthesia operation, postoperative analgesic effect, postoperative muscle strength recovery, and early activity with postoperative nausea and vomiting.

Musculoskeletal disease remains the disturbing issues for people worldwide [27–30]. It has been found that the PENG block can reduce patients' pain during spinal positioning, and the PENG block can reduce the effect on the quadriceps muscle strength so that the patients can get out of bed early. Although heterogeneity was high, sensitivity analyses showed that the results were stable and would not be altered by excluding a particular study. The sensory-motor dissociative effect of the PENG block was significantly better than that of FICB. The effectiveness of the PENG block for analgesia was demonstrated from the results of postoperative pain scores and patients' postoperative opioid consumption, which reduced opioid application due to the inhibition of nociceptive sensitization by better suppression of pain signal conduction from the periphery to the spinal cord at an early stage, based on whether or not the nerve block was used in the control group, and the results of our subgroup analyses showed that the PENG block group was comparable to FICB in reducing patients' postoperative pain. The lack of effect of the PENG block on the incidence of PONV may be due to the importance of PONV prevention and treatment through multimodal programs. It cannot be determined solely by the PENG block alone.

### 4.2. Agreements and Disagreements with Other Studies or Reviews

The results of our meta-analysis showed that the PENG block reduces pain scores during spinal positioning placement and can reduce spinal anesthesia operation time; however, it has the same effect on the effect of pain during spinal positioning compared to FICB. This conclusion is consistent with Mosaffa et al. [16] and controversial with Mao et al. [24]. The results of our meta-analysis are in agreement with the results of Samar Rafik Amin's meta-analysis [31], both of which believe that the PENG block can reduce pain scores during spinal positioning placement. However, this article has limitations. Hua et al. [20] and Jadon et al. [15] did not indicate that the placement time of spinal anesthesia was thirty minutes after the PENG block.

The PENG block does not block the femoral neuromotor branch of the quadriceps muscle, which has a lesser impact on the muscle. The results of our meta-analysis showed that the PENG block can reduce the probability of postoperative hypokinesia when compared with the FICB group, which is consistent with the findings of Aliste et al. [5] and Hua et al. [20]; the PENG block has the same effect on muscle strength as that of the group without the nerve block. Our results concluded that the PENG block could advance the time of patients' first time out of bed walking, further suggesting that the PENG block has less effect on the movement of the quadriceps. The results of our meta-analysis are in agreement with the results of Anwar U. Huda's meta-analysis [32], which concluded that the PENG block caused less risk of motor hindrance. However, they did not mention that patients could be active earlier after surgery.

A review showed that the use of the nerve block is not only better than general analgesia and reduces the risk of postoperative complications, but it also reduces the consumption of opioids [33], which provide adequate analgesia but can cause nausea, constipation, and delirium [34, 35]. Because of this, other analgesic techniques are recommended to reduce opioid consumption in the surgical management of hip fractures. Several RCTs have concluded that the postoperative PENG block reduces postoperative pain in patients, and the results of our meta-analysis showed that the PENG block reduces postoperative pain when compared to the group that did not undergo the block and that the PENG block has a comparable effect on postoperative pain in patients when compared to FICB, a finding that is consistent with the results of a randomized controlled trial by Aliste et al. [5], while Natrajan et al. [17], Mosaffa et al. [16], Senthil et al. [2], Jadon et al. [15], Hua et al. [20], and Mao et al. [24] concluded that postoperative analgesia after the PENG block was superior to FICB. Anwar U. Huda's meta-analysis [32], which included other nerve blocks, concluded that there was no significant difference between the PENG block and other nerve blocks. Ahmed Farag's meta-analysis [36] concluded that there was no significant difference in the pain scores of the PENG block relative to FICB at 6 h, 12 h, 24 h, and 48 h postoperatively; however, they concluded that the PENG block was unable to delay the time to first postoperative remedial analgesia, a conclusion that differed significantly from our results.

### 4.3. Strength and Limitations

This article has the following limitations. First of all, the small amount of literature included in some of the results may have led to imprecise results; the control group should have included some other nerve blocks to fully identify the advantages and disadvantages of the PENG block by comparing it with other nerve blocks. There are only a few articles on spinal anesthesia that reported pain scores during positioning, as well as studies on the operating time of spinal anesthesia, should be added. In addition, we did not explicitly limit the control group treatment in this article, and the different drugs given during anesthesia, the availability of other postoperative analgesia, and the use of different pain scales to record pain scores are all potential factors contributing to heterogeneity.

### 4.4. Implications for Practice

The PENG block can be operated in the supine position [4] and can reduce the pain during spinal positioning, thus increasing the degree of patient cooperation and facilitating the successful implementation of spinal anesthesia. Local analgesia for hip fracture surgery has traditionally used the myofascial block and femoral nerve block, but these blocks can lead to a decrease in postoperative muscle strength; the PENG block, while obtaining analgesic effects similar to those of FICB, has a negligible impact on quadriceps muscle strength because it only blocks sensory nerves such as the femoral nerve, the obturator nerve (ON), and the accessory obturator nerve (AON) [4], which is favorable for the patient's recovery of postoperative muscle strength and earlier mobility. This suggests that the PENG block can be used as a safe and effective regional block technique for patients undergoing hip fracture surgery.

### 4.5. Implications for Research

In the future, the PENG block can be compared with other regional block modalities in detail to discover its advantages and disadvantages fully. To study whether the PENG block will affect the incidence of postoperative lower extremity venous thrombosis, the number of related literature is small, which can be used as the next research direction.

## Figures and Tables

**Table 1:** Characteristics of included studies.

Study	Sample size (*n*)	Type of operation	Experiment group				Control group			
			Sample size (*n*1)	Age (year)	Time of intervention and type of anesthesia	Injectate	Sample size (*n*2)	Age (year)	Time of intervention and type of anesthesia	Injectate
Aliste et al. 2021 [5]	40	Hip arthroplasty	20	56.80 ± 13.00	PENG after operation	20 ml 0.50% adrenalized levobupivacaine	20	59.6 ± 9.2	FICB after operation	40 ml 0.25% adrenalized levobupivacaine
He et al. 2023 [14]	60	Hip fracture surgery	30	72.31 ± 9.24	PENG + DEX (iv.) before SA	20 ml 0.2% ropivacaine 0.2 *μ*g/kg dexmedetomidine (iv.)	30	72.49 ± 8.92	DEX (iv.) before SA	0.2 *μ*g/kg dexmedetomidine (iv.)
Huang et al. 2021 [15]	100	Hip arthroplasty	50	76.50 ± 5.30	PENG before SA	20 ml 0.5% ropivacaine	50	76.8 ± 5.0	SF. before SA	0.15 *μ*g/kg sufentanil (iv.)
Hua et al. 2022 [13]	48	Hip arthroplasty	24	74.00 ± 7.00	PENG before SA	20 ml 0.4% ropivacaine	24	74 ± 8	FICB before SA	30 ml 0.4% ropivacaine hydrochloride
Jadon et al. 2021 [12]	66	Hip fracture	33	70.39 ± 11.45	PENG before SA	—	33	67.87 ± 13.12	FICB before SA	—
Mosaffa et al. 2020 [16]	60	Hip arthroplasty	30	54.00 ± 11.00	PENG before SA	20 ml 0.25% bupivacaine	30	57 ± 8	N/A	N/A
Lin et al. 2022 [17]	60	Total hip arthroplasty	30	68.60 ± 9.50	PENG before SA	20 ml 0.5% ropivacaine	30	68.3 ± 10.9	N/A	20 ml saline
Mao et al. 2022 [18]	120	Hip arthroplasty	60	74.75 ± 6.98	PENG before SA	20 ml 0.375% ropivacaine	60	75.12 ± 7.49	FICB before SA	20 ml 0.375% ropivacaine
Mosaffa et al. 2022 [11]	52	Hip fracture	30	53.00 ± 16.46	PENG before SA	3 ml/kg 0.5% ropivacaine	22	50 ± 13.63	FICB before SA	3 ml/kg 0.5% ropivacaine
Natrajan et al. 2021 [10]	24	Dynamic hip screw fixation or hemiarthroplasty	12	—	PENG before SA	20 ml 0.5% ropivacaine	12	—	FICB before SA	20 ml 0.5% ropivacaine
Pascarella et al. 2021 [19]	60	Total hip arthroplasty	30	66.40 ± 12.40	PENG after SA	20 ml 0.375% ropivacaine	30	66.7 ± 8.6	N/A	N/A
Senthil et al. 2021 [2]	40	Hip fracture	20	53.90 ± 9.90	PENG after operation	30 ml 0.25% levobupivacaine with 4 mg dexamethasone	20	52.5 ± 9.8	FICB after operation	30 ml 0.25% levobupivacaine with 4 mg dexamethasone
Shan et al. 2021 [20]	40	Hip arthroplasty	20	71.40 ± 9.70	PENG before SA	10 ml 0.5% ropivacaine	20	74.8 ± 7.6	SF. before SA	0.1 *μ*g/kg sufentanil (iv.)
Wu et al. 2022 [21]	40	Hip arthroplasty	20	75.80 ± 9.20	PENG before SA	20 ml 0.375% ropivacaine	20	75.7 ± 8.9	SF. before SA	0.2 *μ*g/kg sufentanil (iv.)
Zhang et al. 2022 [22]	80	Hip arthroplasty	40	63.71 ± 6.96	PENG after SA	20 ml 0.4% ropivacaine	40	63.61 ± 7.03	SF. before SA	0.1 *μ*g/kg sufentanil (iv.)

**Table 2:** Assessment of evidence quality.

Quality assessment							No of patients		Effect		Quality	Importance
No of studies	Design	Risk of bias	Inconsistency	Indirectness	Imprecision	Other considerations	PENG	Other analgesic methods	Relative (95% CI)	Absolute		
*PONV*												
8	RCT	Serious^1^	No serious inconsistency	No serious indirectness	No serious imprecision	None	13/242 (5.4%)	20/242 (8.3%)	RR 0.67 (0.34 to 1.32)	27 fewer per 1000 (from 55 fewer to 26 more)	ÅÅÅO moderate	Important
								9.2%		30 fewer per 1000 (from 61 fewer to 29 more)		

*Decreased muscle strength*												
5	RCT	No serious risk of bias	No serious inconsistency	No serious indirectness	Serious^2^	None	0/134 (0%)	9/134 (6.7%)	RR 0.11 (0.01 to 0.86)	60 fewer per 1000 (from 9 fewer to 66 fewer)	ÅÅÅO moderate	Critical
								0%		—		

*VAS during positioning (better indicated by lower values)*												
6	RCT	Serious^1^	Very serious^3^	No serious indirectness	No serious imprecision	None	210	202	—	SMD 1.48 lower (2.35 to 0.62 lower)	ÅOOO very low	Critical

*VAS 6 hour after operation (better indicated by lower values)*												
5	Rct	No serious risk of bias	No serious inconsistency	No serious indirectness	Serious^2^	None	127	127	—	SMD 0.08 lower (0.33 lower to 0.17 higher)	ÅÅÅO moderate	Critical

*VAS 12 hour after operation (better indicated by lower values)*												
5	RCT	No serious risk of bias	Very serious^3^	No serious indirectness	Serious^2^	None	137	137	—	SMD 0.7 lower (1.43 lower to 0.03 higher)	ÅOOO very low	Critical

*VAS 24 hour after operation (better indicated by lower values)*												
5	RCT	No serious risk of bias	Very serious^3^	No serious indirectness	Very serious^2,4^	None	127	127	—	SMD 0.25 lower (1.29 lower to 0.8 higher)	ÅOOO very low	Critical

*VAS 48 hour after operation (better indicated by lower values)*												
3	RCT	No serious risk of bias	No serious inconsistency	No serious indirectness	Serious^2^	None	74	74	—	SMD 0.35 lower (0.67 to 0.02 lower)	ÅÅÅO moderate	Critical

*Postoperative 24-hour morphine consumption (better indicated by lower values)*												
6	RCT	No serious risk of bias	Very serious^3^	No serious indirectness	Serious^2^	None	154	146	—	SMD 1.27 lower (2.19 to 0.35 lower)	ÅOOO very low	Critical

*Postoperative 48-hour morphine consumption (better indicated by lower values)*												
3	RCT	No serious risk of bias	Serious^5^	No serious indirectness	Serious^2^	None	94	94	—	SMD 0.33 lower (0.8 lower to 0.15 higher)	ÅÅOO low	Critical

*Time to first opioid (better indicated by lower values)*												
8	RCT	Serious^1^	Very serious^3^	No serious indirectness	No serious imprecision	None	215	215	—	SMD 1.38 higher (0.41 to 2.34 higher)	ÅOOO very low	Critical

*Spinal anesthesia operation time (better indicated by lower values)*												
2	Rct	No serious risk of bias	Very serious^3^	No serious indirectness	Very serious^2,4^	None	40	40	—	SMD 1.29 lower (2.54 to 0.03 lower)	ÅOOO very low	Critical

*First postoperative walking time (better indicated by lower values)*												
3	RCT	No serious risk of bias	No serious inconsistency	No serious indirectness	Serious^2^	None	120	120	—	SMD 0.9 lower (1.17 to 0.63 lower)	ÅÅÅO moderate	Critical

^1^The included studies that had large biases in randomization, allocation concealment, and blinding. ^2^The sample size of the included study was too small. ^3^*I*^2^ ≥ 75%. ^4^The confidence interval is wide. ^5^50% ≤ *I*^2^ ≤ 75%.

**Table 3:** Subgroup analysis of pain score.

Time	FICB					No block				
	*N*	SMD	95% CI	*P* value	*I* ^2^ (%)	*N*	SMD	95% CI	*P* value	*I* ^2^
Positioning VAS	2	−0.26	−0.93∼0.40	0.44	75	4	−2.15	−3.27∼−1.04	0.0002	91%
6 h VAS	5	−0.08	−0.33∼0.17	0.53	0					
12 h VAS	4	−0.37	−0.85∼0.12	0.14	67	1	−2.15	−2.80∼−1.51	<0.00001	NA
24 h VAS	4	0.29	−0.20∼0.78	0.25	65	1	−2.53	−3.22∼−1.84	<0.00001	NA
48 h VAS	2	−0.20	−0.62∼0.22	0.34	0	1	−0.56	−1.08∼∼−0.04	0.03	NA

**Table 4:** Subgroup analysis of decreased muscle strength.

	FICB					No block				
	*N*	SMD	95% CI	*P* value	*I* ^2^	*N*	SMD	95% CI	*P* value	*I* ^2^
Decreased muscle strength	2	0.11	0.01∼0.86	0.04	0%	3	NA	NA	NA	NA

**Table 5:** Subgroup analysis of opioid consumption in 24 h after surgery.

	FICB					No block				
	*N*	SMD	95% CI	*P* value	*I* ^2^ (%)	*N*	SMD	95% CI	*P* value	*I* ^2^ (%)
Opioid consumption in 24 h after surgery	4	−0.68	−1.30∼−0.77	0.03	75	2	−2.45	−4.07∼−0.84	0.003	91

**Table 6:** Subgroup analysis of first rescue analgesia time after surgery.

	FICB					No block				
	*N*	SMD	95% CI	*P* value	*I* ^2^ (%)	*N*	SMD	95% CI	*P* value	*I* ^2^ (%)
First rescue analgesia time after surgery	3	0.34	−0.83∼1.52	0.57	91	5	1.97	0.99∼2.95	<0.0001	91

## Data Availability

The datasets used and/or analysed during the current study are available from the corresponding author on reasonable request.

## Abbreviations

PENG: Pericapsular nerve group block
FICB: Fascia iliaca compartment block
SMD: Standardized mean difference
RR: Risk ratio.
